# Research of Processing Technology of Longjing Tea with ‘Baiye 1’ Based on Non-Targeted Aroma Metabolomics

**DOI:** 10.3390/foods13091338

**Published:** 2024-04-26

**Authors:** Ruimin Teng, Cun Ao, Haitao Huang, Daliang Shi, Yuxiao Mao, Xuxia Zheng, Yun Zhao

**Affiliations:** Tea Research Institute, Hangzhou Academy of Agricultural Science, Hangzhou 310024, China; tengruimin@tricaas.com (R.T.); aocun123@163.com (C.A.); hthuang309@hotmaill.com (H.H.); sdl7698@126.com (D.S.); m15088717461_1@163.com (Y.M.); xxzheng43@163.com (X.Z.)

**Keywords:** green tea, volatile metabolites, chestnut-like aroma, tea processing

## Abstract

Longjing tea is favored by consumers due to its refreshing and delicate aroma, as well as its fresh and sweet flavor. In order to study the processing technology of Longjing tea with ‘Baiye 1’ tea varieties, solid phase microextraction and gas chromatography–mass spectrometry were used to analyze the volatile components of Longjing tea in different process stages. The results revealed the identification of 275 aroma metabolites in the processing samples of Longjing tea. The sensory evaluation and principal component analysis revealed that the leaves of fresh (XY) and spreading (TF) were different from the leaves of first panning (YQ), second panning (EQ), final panning (HG), and fragrance enhancing (TX). The relative contents of geraniol (1199.95 and 1134.51), linalool (745.93 and 793.98), methyl salicylate (485.22 and 314.67), phenylethyl alcohol (280.14 and 393.98), 2-methylfuran (872.28 and 517.96), 2-butenal (56.01 and 154.60), and 2-hexenal (46.22 and 42.24), refreshing and floral substances in the XY and TF stages, were higher than other stages. The aroma contents of 2-methylfuran, furfural, 2-methyl-1-penten-3-one, 3-hexen-2-one, dodecane, hexanoyl hexanoate, 2,5-dimethyl-pyrazine, and methyl-pyrazine were found to be significantly positively correlated with the intensity of chestnut aroma. In conclusion, this study contributes to a better understanding of the composition and formation mechanism of chestnut-like aroma and provides new insights into the processing technology to improve the quality of albino green tea.

## 1. Introduction

Tea plants contain various secondary metabolites, including tea polyphenols, amino acids, caffeine, catechins, and vitamins. Green tea, a non-fermented tea, is a widely produced and consumed tea in China due to its health benefits [1,2]. Longjing tea, the representative green tea of Zhejiang Province, is favored by consumers for its flat smoothness, refreshing taste, delicate aroma, and sweet flavor [3]. Tea contains a wide variety of aromatic substances. Researchers have extracted and isolated more than 700 types of aroma substances from tea, including alcohols, aldehydes, ketones, acids, esters, phenols, and heterocyclic compounds [4,5]. Despite only accounting for approximately 0.01% of the dry weight of tea, these volatile substances play a crucial role in the sensory properties and quality that attract consumers. The process of producing Longjing tea involves several mechanical steps, including spreading, first panning, second panning, final panning, and fragrance enhancement. The quality of the final product is influenced by various factors such as the variety of tea plant, the tenderness of tea leaves, and the parameters of processing technology [6].

Research and practice have demonstrated that tea processing technology is a crucial factor in determining tea quality [7]. During processing, complex metabolic changes take place, resulting in the production and accumulation of numerous metabolites [8]. Initially, fresh tea leaves only have a grassy scent, but after being picked, the aroma is formed through enzyme-assisted reactions [8]. During the spreading stage, glucoside aroma precursors are hydrolyzed by glucosidase to produce volatile aroma substances such as alcohols, aldehydes, and ketones [9]. Proteolytic enzyme activity increases, causing protein hydrolysis and an increase in the content of soluble protein and free amino acids in the spreading leaves. Fixation (enzyme inactivation) is a key procedure in the formation of the flavor quality of Longjing tea. The tea leaves undergo high-temperature stir frying to inactivate enzymes and form their basic shape and color. The low-boiling point compounds in the fresh leaves that appear grassy are further evaporated [10]. According to Han et al.’s research, enzyme inactivation promotes the degradation of carotenoid and fatty acid aroma precursors in tea, leading to the formation of green tea aroma [11]. The final thermo-chemical step in the formation of the aroma of green tea is drying (fragrance enhancing). Yang et al. detected volatile compounds during the processing of green tea and found that the content of high boiling substances, represented by indole and (Z)-jasmonate, increased during the drying process. Additionally, a large amount of dimethyl sulfur was produced during this process [12]. Ni et al. discovered that elevating the temperature of the tea final-panning machine facilitated the production of linalool and terpene alcohols with floral and fruity aromas, as well as pyrazine compounds with roasted aromas in tea [13].

The aroma of tea is an important factor in determining its quality and taste [14,15]. According to traditional sensory evaluation, green tea aroma can be divided into caramel, bean, high heat fired, clear and refreshing, flowery, fruity, and chestnut aromas [16,17]. Mizukami et al. identified 2-ethyl-3,5-dimethyl-pyrazine, tetramethyl-pyrazine, and 2,3-diethyl-5-methyl-pyrazine as key compounds in roasted aromatic green tea [18]. According to Wang et al’s gas chromatography-olfactometry (GC-O) analysis and odor activity values (OAVs) calculation, the primary aroma compounds in bean-flavored green tea were butyl acetate, phenyl-acetaldehyde, geraniol, and dimethylbutyraldehyde. Meanwhile, the primary aroma compounds in tender green tea were (-)-limonene, linalool, and dimethylsulphur [19]. Bayesian stepwise discriminant analysis was used to screen β-ionone, phytol, linalool, and other compounds to distinguish chestnut aroma from non-chestnut aroma tea samples. Zhang et al. used HS-SPME combined with GC×GC-TOFMS technology and identified isobutyraldehyde, hexanal, heptanal, nonanal, decanal, phenyl-acetaldehyde, linalool, 1-octen-3-ol, 1-octen-3-one, β-ionone, p-cymene, and ethyl hexanoate as the key components of chestnut green tea [20]. 

Previous reports on Longjing tea have primarily focused on the production process and taste of green leaf varieties. However, there is limited understanding of the volatile and nonvolatile metabolites of Longjing tea. ‘Baiye 1’ is a high-quality tea plant variety in Zhejiang Province. Its spring young buds and leaves are jade white in color with light green veins and have a high amino acid content, making it suitable for producing green tea. To our knowledge, no reports exist on volatile metabolomics studies of Longjing tea with ‘Baiye 1’ varieties. This study used non-targeted metabolomics detection technology to analyze volatile metabolites in samples from each processing process and to screen and identify differential metabolites. The results provide a reference for further understanding of the quality formation mechanism of Longjing tea. 

## 2. Materials and Methods

### 2.1. Reagents and Materials 

The materials used in this experiment were tea plant cultivar ‘Baiye 1’ and planted in Hangzhou area (Hangzhou, China). In March, the samples of tea plant cultivar ‘Baiye 1’ with the tenderness of one bud and one leaf were collected for the subsequent assays.

**Figure 1:**
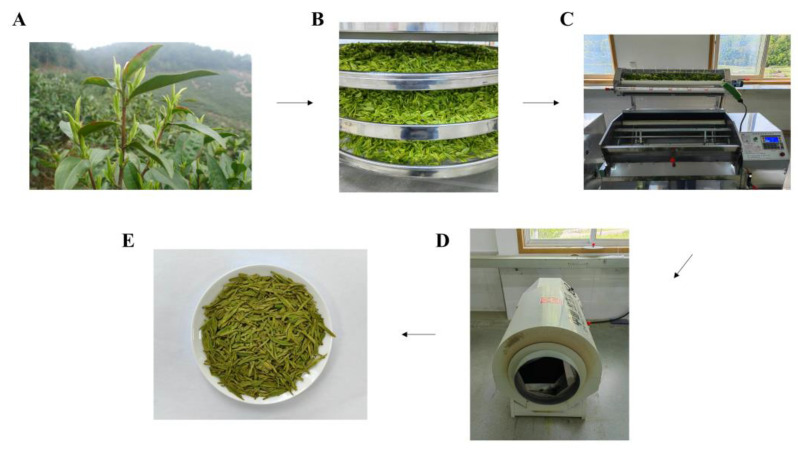
Schematic diagram of Longjing tea leaf processing. (**A**) XY; (**B**) TF; (**C**) HG; (**D**) TX; and (**E**) Longjing tea. XY indicates fresh leaves, TF indicates spreading fresh leaves, HG indicates final panning, and TX indicates fragrance enhancing.

The green tea was processed using an automatic roasting machine for flat tea (6CCB-100ZD, Hengfeng Technology Development Co., Ltd., Shaoxing, China) and then dried and aromatized using a tea final-panning machine (6CH-2.0A, Zhejiang Hongwuhuan Tea Equipment Co., Ltd., Quzhou, China). After the fresh leaves (XY) were spreading (TF) for 16 h, the weight of the first panning leaf (YQ) was 100 g, and it was stir-fried for 110 circles at a temperature of 190 °C. The weight of the second panning leaf (EQ) was 60 g, and it was stir-fried for 90 circles at a temperature of 160 °C. Further, final panning (HG) occurred at a temperature of 100 °C for 20 min and fragrance enhancing (TX) at a temperature of 135 °C for 10 min (Figure 1). 

Samples for determining taste components and conducting sensory evaluations: Fresh and spread leaves were placed thinly (1 cm) in a microwave oven and microwaved at high heat for 2 min for the first time. After cooling down, they were microwaved at low heat for 1 min. Finally, they were baked at 60 °C for 1 h until fully dry. First and second panning tea leaves were baked directly at 60 °C for 0.5 to 1 h until fully dry.

Samples for analyzing aroma components: The tea samples of each processing step were rapidly frozen in liquid nitrogen for 10 min, then stored at −80 °C. Prior to analysis, the samples were freeze-dried.

### 2.2. Chemicals

Gallocatechin (GC, ≥99% pure by HPLC), catechin (C, ≥99%), epigallocatechin (EGC, ≥99%), epicatechin (EC, ≥99%), epigallocatechin gallate (EGCG, ≥99%), gallocatechin gallate (GCG, ≥99%), catechingallate (CG, ≥99%), epicatechin gallate (ECG, ≥99%), caffeine, (≥99%), and theanine (≥99%) standards were purchased from Sigma-Aldrich (Shanghai, China). Gallic acid (98%, HPLC-grade), Folin phenol (98%), acetonitrile (99.9%, HPLC-grade), anthrone (97%), and acetic acid (99.7%, HPLC-grade) were obtained from Sinopharm MEDICINE Holding Co., Ltd. (Shanghai, China). Ninhydrin (99.9%) and glutamic acid (99%, HPLC-grade) were obtained from Aladdin biochemical technology Co., Ltd. (Shanghai, China). Ethyl decanoate (99%, HPLC-grade) and amino acid mixing (99%, HPLC-grade) standards were purchased from HITACHI (Shanghai, China). 

### 2.3. Detection of Volatile Aroma Components

Non-targeted volatile metabolomics analysis was commissioned by Shanghai Luming Biotechnology Co., Ltd. Solid phase microextraction (SPME, Basel, Switzerland) was used to extract volatile components from tea according to An et al.’s approach [21]. The sample (1 g) was mixed with 20 μL of ethyl decanoate (internal standard, 0.02 mg/mL in ethanol) and then transferred to a 20 mL headspace vial. The sample was subjected to headspace extraction at a constant temperature of 50 °C using an SPME fiber (CTC Analytics AG, Basel, Switzerland) (50/30 μm DVB/CAR on PDMS) for 30 min. The extracted sample was desorbed at 250 °C for 5 min and analyzed using HS-SPME-GC–MS (gas chromatography–mass spectrometry, Agilent, Santa Clara, CA, USA). A DB-WAX gas column (30 m × 0.25 mm × 0.25 μm, Agilent J&W Scientific, Folsom, CA, USA) was used for GC analysis. The inlet temperature was 260 °C, and the solvent was delayed for 1.5 min without shunt injection. The temperature was maintained at 40 °C for 3 min, then increased to 220 °C at a rate of 5 °C per minute and retained for 5 min. The helium carrier gas flow rate was 1.0 mL/min, and the mass spectrometer operated in electron ionizati (EI) mode at an ionization voltage of 70 eV. The ion source and transmission line temperatures were 250 °C and 150 °C, respectively, and the mass spectrum was obtained in full scan mode.

### 2.4. Qualitative and Quantitative Analyses of Aroma Components in Tea

The obtained GC–MS spectra were compared with the standard mass spectra provided by computer retrieval and the NIST mass spectrometry library. Aromatic substances were identified by comparing peak type and peak emergence time with published mass spectra [21]. 

The internal standard was used to control the quality of the data by removing internal standard peaks and false positive peaks (including noise, column loss, and derivatized physical and chemical reagent peaks) and deleting ion peaks with missing values (>50% for each group). Any remaining missing values (0 value) were replaced by half of the minimum value. For each sample, the total peak area was normalized by all peak signal intensities (peak areas) to calculate the relative content of each aroma.

### 2.5. Determination of Chemical Constituents in Tea Leaves

The samples were extracted according to the method described by Cui et al. [22]. Briefly, approximately 3 g of dry weight of samples was extracted in 500 mL of water and centrifuged for 10 min at 3500 rpm. Subsequently, the supernatant was filtered through a 0.22 μm aqueous phase membrane and then reserved for further analysis. The determination of catechin monomers and caffeine was conducted using a high-performance liquid chromatography (HPLC, Agilent 1100, Santa Clara, CA, USA) system with an ultraviolet detector. Chromatographic separation was performed using a Hypsial ODS C_18_ column (4.6 mm × 250 mm, 5 μm). The mobile phase A consisted of 2% (*v*/*v*) acetic acid, while mobile phase B was 100% (*v*/*v*) acetonitrile. The flow rate was set to 1 mL/min, the detection wavelength was 280 nm, and the column temperature was 35 °C. The gradient elution procedure involved a change in mobile phase A from 93.5% to 92% within 0–12 min, from 92% to 85% in 12–16 min, from 85% to 15% in 16–20 min, followed by a return to the initial state of mobile phase A (93.5%) in 20–30 min, and equilibration for 5 min. 

The total content of tea polyphenols was determined using the Folin phenol colorimetric method. A total of 5 mL 10% (*v*/*v*) of Folin phenol reagent was added into 1 mL of the supernatant solution, which was then shaken well and left to react for 8 min. Subsequently, 4 mL of 7.5% sodium carbonate solution was added, and the mixture was then left for 1 h at room temperature. The absorbance of each sample was determined by spectrophotometer at 765 nm with water as a control. The total amount of free amino acids was determined using ninhydrin colorimetry, and the amino acid components were determined using a high-speed amino acid analyzer (LA 8080 SAAYA, Hitachi, Tokyo, Japan), according to GB/T 8314-2013 [23]. The soluble sugar content was determined using anthrone colorimetry, as described in a previous study [24]. 

### 2.6. Sensory Evaluation

In accordance with the tea sensory evaluation method (GB/T 23776-2009) [25], 11 senior tea assessors or above conducted a coded evaluation of aroma quality. The subfactors of aroma quality were divided into overall aroma intensity (richness), chestnut aroma, floral aroma, fragrance, tender aroma, fire quality, and sweet aroma intensity. Each factor was scored individually on a 9-point scale, where a score of 3 indicates ‘presence’ and 6 indicates ‘prominence’.

The taste quality was evaluated based on several subfactors, including concentration (thickness and stimulation), bitterness, astringency, sweetness, freshness, and smoothness. Each subfactor was rated individually on a 9-point scale, with 3 points indicating ‘moderate’ and 6 points indicating ‘strong’. 

### 2.7. Statistical Analysis

The experimental data were statistically analyzed by SPSS17.0 (SPSS Inc., Chicago, CA, USA), and figures were drawn by GraphPad prism. Each sample was performed with three biological replicates. Principal component analysis (PCA) based on Bray–Curtis distance was performed using R package to assess aroma differences during tea processing. Model differences in the relationship between aroma metabolite expression and processing were analyzed by visualization using partial least squares-discriminant analysis (PLS-DA). Differences in metabolite composition between processing treatments were analyzed using *t*-test values for pairwise comparisons (significant differences at *p* < 0.05, VIP > 1). Correlation analysis (Pearson correlation coefficients) was carried out using Origin 8.0 (MicroCal, Northampton, MA, USA) to evaluate the correlation between the aroma values of the chestnut aroma and the relative content of the aroma compounds at each stage of processing. A correlation heatmap was plotted on the obtained values. One-way analysis of variance (ANOVA) (*p* < 0.05) was used to detect the differences in representative compounds during processing stage.

## 3. Results and Discussion

### 3.1. Aroma Quality Analysis

**Figure 2:**
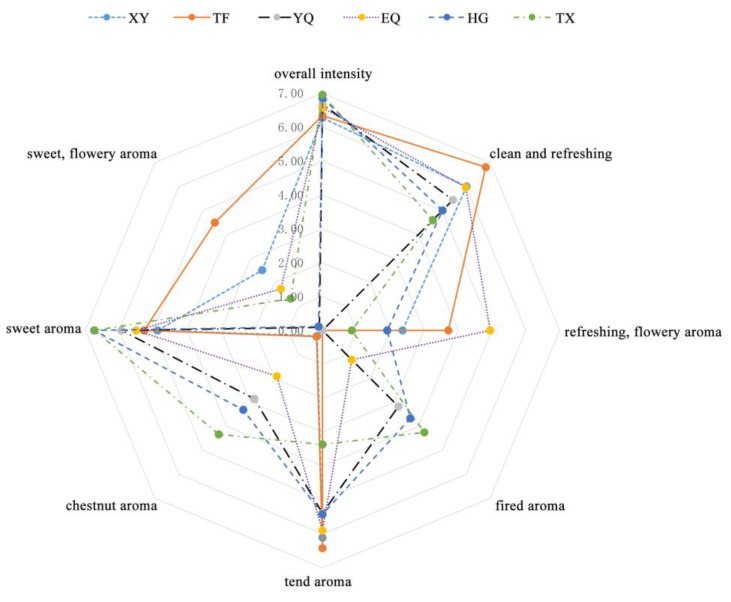
Odor profiles of the infusions of green tea Longjing. XY indicates fresh leaves, TF indicates spreading fresh leaves, YQ indicates first panning leaves, EQ indicates second panning leaves, HG indicates final panning leaves, and TX indicates fragrance enhancing leaves.

Sensory evaluation was used to evaluate the aroma of Longjing tea leaves at each processing step. The results are shown in Figure 2; the overall intensity of the tea aroma increased slightly as processing progressed. However, the intensity of fragrance, floral, and tender aromas gradually weakened, with the highest intensity observed in the TF stage. Conversely, the intensity of fire, sweet, and chestnut aromas gradually increased, with the highest intensities observed in the HG and TX stages. 

### 3.2. Multivariate Statistical Analysis of Aroma Metabolites during Tea Leaf Processing

**Figure 3:**
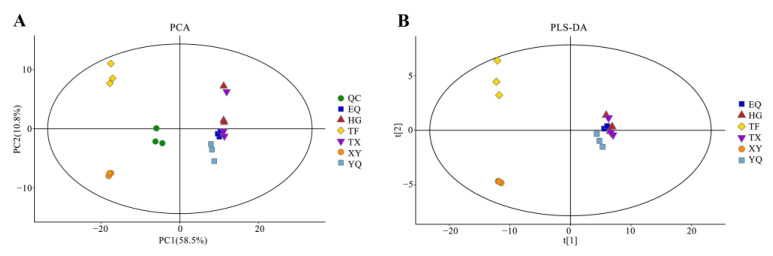
Multivariate statistical analysis of tea aroma components in six different processing processes. (**A**) PCA score plot of relative differences in aroma metabolites of six processing processes of tea; (**B**) PLS-DA score plot of relative differences in aroma metabolites of six processing processes of tea.

PCA and PLS-DA have been widely used in food research as multivariate analysis methods [26,27]. To investigate aroma metabolites significantly associated with tea processing, PCA and PLS-DA models were applied to GC–MS datasets. The chromatography and quality detection systems were validated using QC samples. The PCA score plots showed tightly clustered QC samples, with the location of the QC samples close to the origin of the coordinates. These results confirmed the repeatability and stability of the analytical method. In the PCA score plot (Figure 3A), the first and second principal components explained 58.5% and 10.8% of the variation, respectively. The phenotypes of the six processes were well distinguished, with all repetitions of each process clustered together. The TF and XY stages were separated from the other processes and clustered in the upper right and lower right quadrants of the score plot, respectively. YQ, EQ, HG, and TX were clustered in the upper left and lower left quadrants of the score chart, respectively.

PLS-DA was used to distinguish the differences in metabolite composition among the different processing steps. The PLS-DA model fit parameters were R2X = 0.87, explanatory rate R2Y = 0.579, and predictability Q2 = 0.41 (Figure 3B). The model demonstrates a strong ability for cumulative interpretation and prediction and is stable and reliable. The PCA and PLS-DA score plots showed significant differences among samples from distinct processing methods, indicating that tea aroma metabolites were influenced by processing conditions.

### 3.3. Metabolic Changes in Aroma Substances during Tea Processing

**Figure 4:**
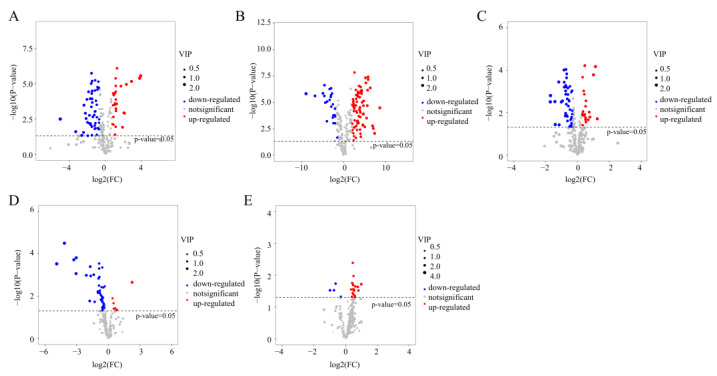
Volcano plot of the number of differential metabolites of aroma in Longjing processing. (**A**) XY vs. TF; (**B**) TF vs. YQ; (**C**) YQ vs. EQ; (**D**) EQ vs. HG; and (**E**) HG vs. TX. XY indicates fresh leaves, TF indicates spreading fresh leaves, YQ indicates first panning leaves, EQ indicates second panning leaves, HG indicates final panning leaves, and TX indicates fragrance enhancing leaves.

The aim of this study was to investigate the impact of each stage of green tea processing on aroma substances and identify key differences between the processes. Aroma metabolomics analysis was conducted on samples from Longjing tea processing, resulting in the identification of 275 differential aroma metabolites. A total of 76 differential metabolites were annotated at the XY/TF stages (27 of up-regulation, 49 of down-regulation), 104 differential metabolites were recorded at the TF/YQ stages (80 of up-regulation, 24 of down-regulation), 67 differential metabolites were observed at the YQ/EQ stages (22 of up-regulation, 45 of down-regulation), 51 differential metabolites were observed at the EQ/HG stages (7 of up-regulation, 44 of down-regulation), and 23 differential metabolites were observed at the HG/TX stages (19 of up-regulation, 4 of down-regulation) (Figure 4A–E). 

### 3.4. Metabolite Profiling Analysis

**Figure 5:**
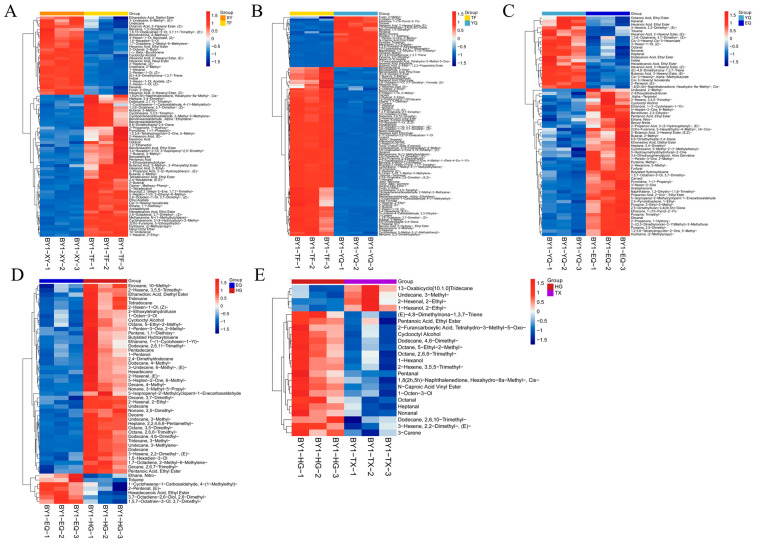
Heatmap of identified differential metabolites of aroma by different processing processes in Longjing tea. (**A**) XY vs. TF; (**B**) TF vs. YQ; (**C**) YQ vs. EQ; (**D**) EQ vs. HG; and (**E**) HG vs. TX. XY indicates fresh leaves, TF indicates spreading fresh leaves, YQ indicates first panning leaves, EQ indicates second panning leaves, HG indicates final panning leaves, and TX indicates fragrance enhancing leaves.

To visually demonstrate the differences in metabolite expression during processing, we performed hierarchical clustering analysis on the expression of all significantly different metabolites separately (Figure 5 and Table S1).

As shown in Figure 5A, the relative content of 1-hexanol, hexanal (hexanoic acid, ethyl ester), hexanoic acid, 3-hexenyl ester, (Z)-2-hexen-1-ol, 1,5-hexadien-3-ol, 2-methyl-undecane, and (Z)-2-penten-1-ol aroma metabolites during the XY stage was 94.16, 44.97, 34.97, 30.85, 22.73, 20.34, 19.84, and 18.67, respectively, higher than those in the TF stage. However, the relative content of acetaldehyde, 2-butenal, (2,6-octadien-1-ol, 3,7-dimethyl-, (Z)-), (benzeneacetaldehyde, alpha-ethylidene-), (2,6-octadienal, 3,7-dimethyl-, (Z)-) (2,4-hexadienal, (E,E)-), (cyclopentanecarboxaldehyde, 2-methyl-3-methylene-), benzaldehyde, hexanoic acid, ethyl acetate, (hexadecanoic acid, ethyl ester), (3-methyl-butanal), 2-methyl-butanal, (heptane, 2,4-dimethyl-), (ethane, 1,1-diethoxy-), and benzeneacetaldehyde increased significantly in the TF stage.

It has been reported that fresh leaves contain higher levels of grass gases such as cis-3-hexenol, trans-2-hexenal, hexanal, and isoamyl alcohol [28]. In this study, compared with fresh leaves, the content of hexanal decreased by 45% and hexanol decreased by 59% in spreading leaves. As water is lost during spreading, it promotes the emission of a grassy scent, which leads to the formation of aromatic substances with a pleasant and fragrant aroma. 

The spread of fresh tea leaves is conducive to the hydrolysis of proteins, lipids, pigments, polysaccharides, glycosides, and other substances by hydrolases and promotes the accumulation of tea aroma precursors [29]. Enzyme inactivation is crucial for the formation of green tea’s ‘green leaves and clear soup’ quality. In addition to passivating enzyme activity to ensure the basic quality of green tea, it also plays an important role in the formation of aroma.

As shown in Figure 5B, the relative content of geraniol (1314.51), linalool (793.98), phenylethyl alcohol (393.98), trans-linalool oxide (furanoid) (365.20), methyl salicylate (314.67), acetaldehyde, beta-myrcene, 2-butenal (154.60), (5,9-undecadien-2-one, 6,10-dimethyl-, (E)-), carveol, benzyl alcohol, trans-.beta.-ocimene, (Z)-3-hexen-1-Ol, (E)-2-hexenal (42.24), 1-hexanol, (2,6-octadien-1-ol, 3,7-dimethyl-, (Z)-), hexanal, D-limonene, (benzeneacetaldehyde, .alpha.-ethylidene-), and (2,6-octadienal, 3,7-dimethyl-, (Z)-) in the TF stage is higher than that in the YQ stage. However, the relative content of some aroma metabolites in the YQ stage is higher than that in the TF stage, such as for 2-methylfuran, methyl alcohol, (butanoic acid, 2-ethyl-, 1,2,3-propanetriyl ester), (pentane, 1,1-diethoxy-), pentanal, (N-caproic acid vinyl ester), (2-furancarboxylic acid, tetrahydro-3-methyl-5-oxo-), (3,5-octanedione, 2,2,4,7-tetramethyl-), (benzene, 1,3-dimethyl-), (hexanoic acid, anhydride), toluene, nonan-4-yl acetate, (E)-4,8-dimethylnona-1,3,7-triene, 2,5-dimethyl-pyrazine, 3-hexen-2-one, (hexanoic acid, 2-hexenyl ester, (E)-), and methyl-pyrazine.

Most of the volatile compounds formed during processing are due to mechanical damage and high temperatures of the leaves, which promote enzymatic, hydrolytic, and thermal cleavage reactions of volatile compounds. In this paper, the content of linalool, (Z)-3-hexen-1-ol, hexanal, and other aromatic substances with low boiling points were significantly decreased in the YQ stage. The content of 2-methylfuran, N-caproic acid vinyl ester, and 2, 5-dimethyl-pyrazine increased significantly in the YQ stage. 

As shown in Figure 5C, the relative content of some aroma metabolites in the YQ stage is higher than that in the EQ stage, for example, (hexanoic acid, 3-hexenyl ester, (Z)-), (1,8(2h,5h)-naphthalenedione, hexahydro-8a-methyl-, cis-), (cis-3-hexenyl-.alpha.-methylbutyrate), (hexadecanoic acid, ethyl ester), nonanal, (butanoic acid, 3-hexenyl ester, (E)-), heptanal, cis-3-hexenyl Isovalerate, toluene, (hexanoic acid, ethyl ester), (octanoic acid, ethyl ester), and hexanal. However, the relative content of some aroma metabolites in the EQ stage is higher than that in the YQ stage, such as for carveol, (1,5,7-octatrien-3-ol, 3,7-dimethyl-), 2,4-dimethyl-heptane, (ethanedioic acid, diethyl ester), nitro- ethane, 3-hexen-2-one, 2,5-dimethyl-pyrazine, 2-methyl- butanal, 2-methyl-undecane, (5-hepten-2-one, 6-methyl-), and (propanoic acid, 2-oxo-, ethyl ester). Glucose and amino acids can produce aromatic substances such as pyrazine, pyrrole, and pyran when heated and dried [30]. In this paper, during the YQ to EQ stage, the contents of pyrazine, pyrrole, and pyran substances increase, presenting a pleasant baking aroma.

As shown in Figure 5D, the relative content of some aroma metabolites in the EQ stage is higher than that in the HG stage, for example, (3,7-octadiene-2,6-diol, 2,6-dimethyl-), nitro- ethane, (1-cyclohexene-1-carboxaldehyde, 4-(1-methylethyl)-), toluene, (E)-2-pentenal, (hexadecanoic acid, ethyl ester), and (1,5,7-octatrien-3-ol, 3,7-dimethyl-). However, the relative content of some aroma metabolites in the HG stage is higher than that in the EQ stage, such as 1,1-diethoxy-pentane, 3,5-dimethyl- octane, 2,2,4,6,6-pentamethyl- heptane, dodecane, (ethanedioic acid, diethyl ester), 2,6,6-trimethyl-octane, 1,5-hexadien-3-ol, 4,6-dimethyl- dodecane, pentadecane, 1-pentanol, 3-methyl-undecane, decane, 1-octen-3-ol, (5-hepten-2-one, 6-methyl-), and 2,6,7-trimethyl-decane. During the HG stage, the contents of dodecane, decane, pentadecane, hexadecane, tetradecane, and other substances increased significantly. These substances have been identified as the components of chestnut aroma [16].

As shown in Figure 5E, the relative content of some aroma metabolites in the HG stage is higher than that in the TX stage, for example, (2-furancarboxylic acid, tetrahydro-3-methyl-5-oxo-), N-caproic acid vinyl ester, pentanal, (1,8(2h,5h)-naphthalenedione, hexahydro-8a-methyl-, cis-), (octane, 2,6,6-trimethyl-), (dodecane, 4,6-dimethyl-), 1-octen-3-ol, nonanal, (2-hexene, 3,5,5-trimethyl-), heptanal, 1-hexanol, and (octane, 5-ethyl-2-methyl-). However, the relative content of some aroma metabolites in the HG stage is higher than that in the EQ stage, such as for (undecane, 3-methyl-), (1-hexanol, 2-ethyl-), (2-hexenal, 2-ethyl-), and (13-oxabicyclo[10.1.0]tridecane).

### 3.5. Characteristic Aroma Component

The plant tissue of fresh leaves contains only small amounts of free volatile compounds [15,31]. Tea aroma components are formed through four main pathways: oxidative degradation of carotenoids, oxidative degradation of fatty acids, hydrolysis of glycosides, and Maillard reaction [32].

The difference in aroma component content in samples at different processing stages was compared and analyzed, and those with high relative content and a strong correlation with the main odorant components were screened, as shown in Table S2. The relative content of more than 4‰ in fresh leaves includes 2-butenal, (E)-2-hexenal, (Z)-3-hexen-1-ol, (E)-6,10-dimethyl-5,9-undecadien-2-one, acetaldehyde, benzyl alcohol, benzoyl isothiocyanate, hexanal, carveol, and trans-β-ocimene, which are mostly flowery or refreshing substances. The above components are the main aroma-enhancing substances of fresh tea leaves.

The aroma of tea transforms from fresh to chestnut, sweet, and fire. The relative contents of seven aroma substances, including geraniol, linalool, and its oxides I, methyl salicylate, phenylethyl alcohol, 2-methylfuran, and β-myrcene, were more than 1% in fresh and spread leaves, in which the rest of them were flowery or refreshing aroma substances except for 2-methylfuran. After the YQ stage, their contents were significantly reduced, with most of them reduced by more than 90%. Wang et al. revealed that linalool, nonanal, trans-β-ionone, hexanoic acid (Z)-3-hexenyl ester, ethylbenzene, naphthalene, and 2-pentylfuran were important components in the formation of green tea fragrance aroma quality [33]. During the processing of Longjing tea, linalool and geraniol produce aroma substances under the reaction of glucosidase, which play an important role in the formation of the light and refreshing floral aroma of Longjing tea and are one of the main aroma-presenting substances, which determines the aroma quality of tea to a certain extent.

During thermal processing, 2-methylfuran, tetrahydro-3-methyl-5-oxo-2-furancarboxylic acid, cis-hexahydro-8a-methyl-1,8(2h,5h)-naphthalenedione, 2,2,4,7-tetramethyl-3,5-octanedione, 2-ethyl-butanoic acid,1,2,3-propanetriyl ester, 2,2,4,6,6-pentamethyl- heptane, O-methyl-hydroxylamine, N-caproic acid vinyl ester, dodecane, and 3,5-dimethyloctane were significantly increased, and the relative contents were higher than 1‰. During the tea rolling process, the quantity and content of aldehydes, ketones, and esters increased greatly due to enzymatic oxidation. 3,5-Octanedione, 2,2,4,7-Tetramethyl-.

Some studies have shown that chestnut aroma is generally considered to be closely related to pyrazine, pyrrole, and other caramel aroma substances generated by thermophysical and chemical reactions. In this paper, 2-((3,3-dimethyloxiran-2-yl) methyl)-3-methylfuran, tetrahydro-3-methyl-5-oxo-2-furancarboxylic acid, 1-hydroxy-2-propanone, 2,5-dimethylfuran-3,4(2h,5h)-dione, butylated hydroxytoluene, hexanoyl hexanoate, nonan-4-yl acetate, 3-methylundecane, 3-hexen-2-one, 2,3-dihydrobenzofuran, 2-methyl-1-penten-3-one, trimethyl-pyrazine, 2,5-dimethyl- pyrazine, methyl-pyrazine, and furfural increased by more than 10 times, which may be closely related to the baking aroma and roasted flavor of tea.

**Figure 6:**
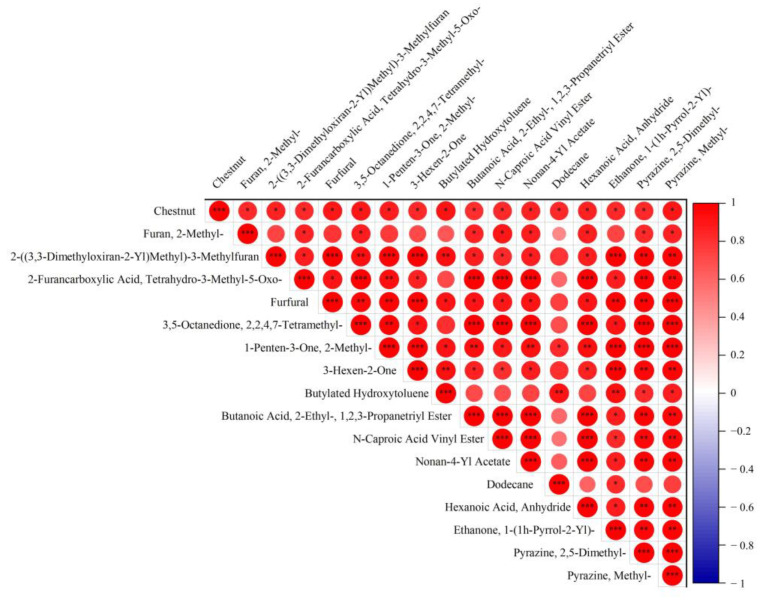
Correlation analysis of chestnut aroma substances. *, **, and *** indicate significance at *p* < 0.05, *p* < 0.01, and *p* < 0.001, respectively.

By correlating the differential components with the intensity of chestnut aroma, the results showed that 2-methylfuran, tetrahydro-3-methyl-5-oxo-2-furancarboxylic acid, 2-((3,3-dimethyloxiran-2-yl)methyl)-3-methylfuran, furfural, 2,2,4,7-tetramethyl-3,5-octanedione, 2-methyl-1-penten-3-one, 3-hexen-2-one, butylated hydroxytoluene, 2-ethyl-butanoic acid-1,2,3-propanetriyl ester, N-caproic acid vinyl ester, nonan-4-yl acetate, dodecane, hexanoyl hexanoate, 1-(1h-pyrrol-2-yl)-ethanone, 2,5-dimethyl-pyrazine, and methyl-pyrazine showed a statistically significant correlation with the intensity of chestnut aroma, which may be the main aroma-enhancing constituents of chestnut aroma (Figure 6).

### 3.6. Changes in Flavor Substances during Tea Processing

**Figure 7:**
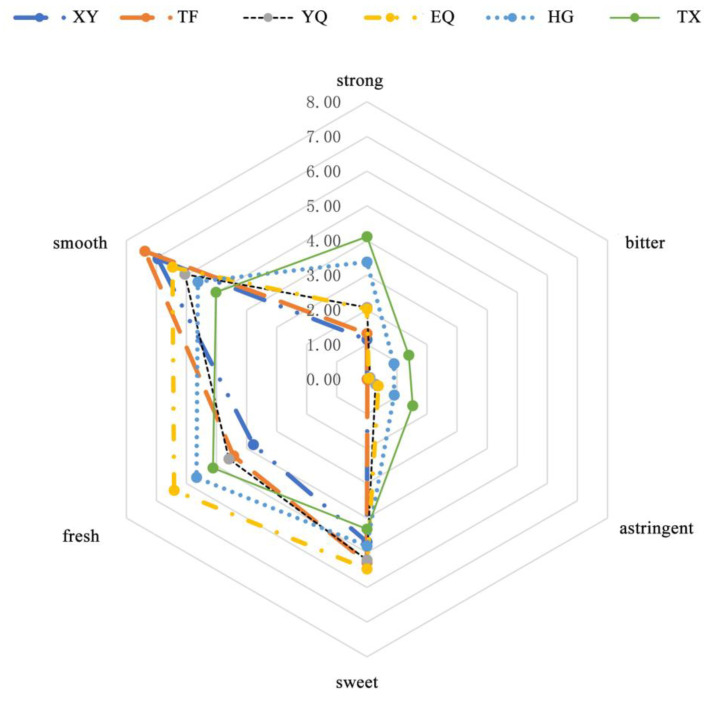
Flavor profiles of the infusions of green tea Longjing. XY indicates fresh leaves, TF indicates spreading fresh leaves, YQ indicates first panning leaves, EQ indicates second panning leaves, HG indicates final panning leaves, and TX indicates fragrance enhancing leaves.

The taste quality of tea samples at different processing stages was evaluated, as shown in Figure 7. The sweetness, freshness, and smoothness of the tea soup increased after the TF stage, which may be related to the decrease in the phenol-ammonia ratio [34,35]. The concentration, bitterness, and astringency of the tea soup flavor increased with the thermal processing, while the smoothness decreased. This was due to the increase in the concentration of water extract and tea polyphenols in the tea soup, especially at the HG stage. However, the sweetness and freshness of the tea soup exhibited a pattern of increase followed by decrease, with the highest values observed during the EQ stage. This suggested that bitterness and astringency had a significant inhibitory effect on sweetness and freshness.

### 3.7. Metabolic Changes in Taste Substances during Tea Processing

**Figure 8:**
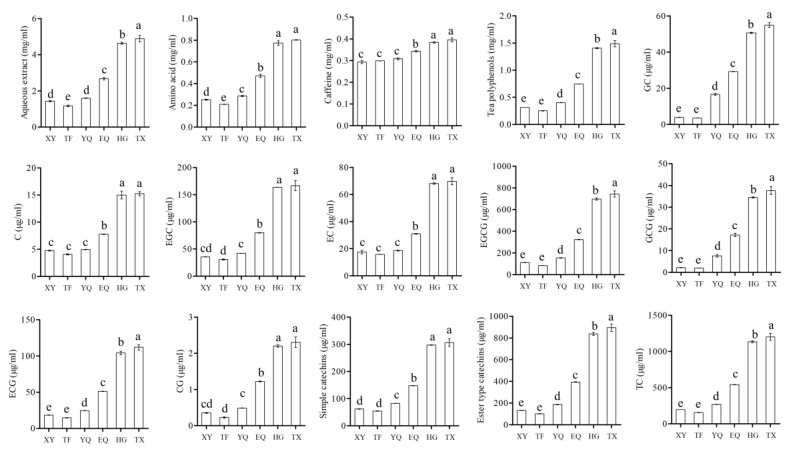
Changes in representative compounds in tea during different processing processes. Different lowercase letters indicate significant differences at *p* < 0.05. GC, gallocatechin; C, catechin; EGC, epigallocatechin; EC, epicatechin; EGCG, epigallocatechin gallate; GCG, gallocatechin gallate; CG, catechingallate; ECG, epicatechin gallate; simple catechins (GC + C + EGC + EC); ester-type catechins (EGCG + GCG + CG + ECG); XY indicates fresh leaves; TF indicates spreading fresh leaves; YQ indicates first panning leaves; EQ indicates second panning leaves; HG indicates final panning leaves; and TX indicates fragrance enhancing leaves.

The concentration of flavor components in tea brewed from tea samples at different stages of Longjing tea processing was analyzed. As shown in Figure 8 and Table S3, the concentration of tea polyphenols, amino acids, water extracts, and other taste components in the tea soup increased continuously with the thermal processing, particularly in the HG stage, followed by the EQ stage, and the least increase in the TX stage. The concentration of various flavor components in the tea samples was lowest during the TF stage, where the water extract and amino acids concentrations were significantly lower than the XY stage. This may be due to the higher water content of the fresh leaves; in the microwave process and fixing of samples, the materials are more likely to be puffed up; the cell and tissue fragmentation rates were higher than that of the spreading stalled leaves; and the flavor components were more likely to be leached out and lead to these results [34,36].

**Figure 9:**
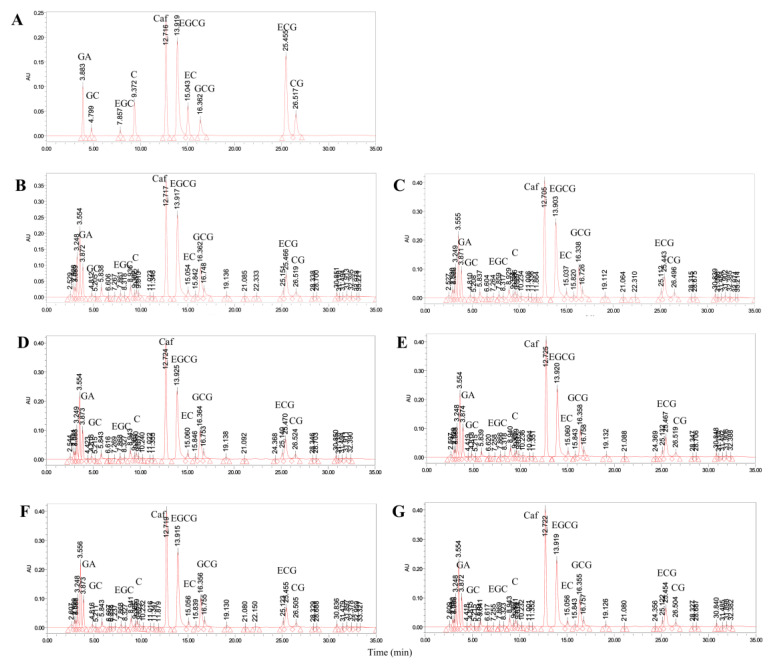
HPLC profiles of catechins in tea during different processing processes. (**A**) Standard samples of eight catechins; (**B**) XY stage; (**C**) TF stage; (**D**) YQ stage; (**E**) EQ stage; (**F**) HG stage; and (**G**) TX stage. GC, gallocatechin; C, catechin; EGC, epigallocatechin; EC, epicatechin; EGCG, epigallocatechin gallate; GCG, gallocatechin gallate; CG, catechingallate; ECG, epicatechin gallate; XY indicates fresh leaves; TF indicates spreading fresh leaves; YQ indicates first panning leaves; EQ indicates second panning leaves; HG indicates final panning leaves; and TX indicates fragrance enhancing leaves.

During tea processing, the concentration of tea polyphenols increased significantly while caffeine increased to a lesser extent. This suggests that cellular fragmentation has a greater impact on the dissolution of tea polyphenols. Additionally, ester-type catechins increased more than simple catechins during the process of EQ to HG stage, as shown in Figure 8 and Figure 9. After the EQ stage, the concentration of CG increased by 2.48 times that of the YQ stage, while the concentration of C increased the least. After the HG stage, the concentration of EC increased proportionally the most, while the concentration of GC increased proportionally the least. The amino acid composition of tea samples at different stages was significantly different, with increased amino acid concentrations at the YQ, EQ, HG, and TX stages compared to the TF stage. The concentration of umami amino acids, such as L-aspartic acid, glutamic acid, and theanine, as well as sweet amino acids, such as L-serine, alanine, and threonine, increased at higher multiples from the YQ to EQ stages than that from the EQ to HG stages. Bitter amino acid concentration increased the highest, and sweet amino acid concentration increased the lowest (Table S3).

The dry matter ratios of tea flavor components at each stage were analyzed, and the results are shown in Figures S1 and S2 and Table S4. The proportions of amino acids, tea polyphenols, and caffeine increased significantly, while the proportions of soluble sugar and water extract decreased significantly after the fresh leaves were spread. With thermal processing, the levels of tea polyphenols, caffeine, total catechins, and ester-type catechins such as EGCG, GCG, ECG, and CG decreased gradually, and the total soluble sugars remained relatively stable. The total free amino acids decreased during the TX stage, which might be involved in the Maillard reaction [32,37].

The amino acid composition of tea samples at different stages was significantly different. The umami amino acids in tea accounted for more than 80% of the total amino acids, with theanine being the most important, accounting for about 70% of the total amino acids. The amino acids with more than 1‰ content are theanine, arginine, glutamic acid, aspartic acid, serine, and cystine, among which arginine is a bitter amino acid, serine is a sweet amino acid, and theanine, glutamic acid, and aspartic acid are umami amino acids. During the TF stage, the bitter amino acids showed the largest percentage increase of 32.1%, with the most significant increase occurring in very low levels of valine, isoleucine, tyrosine, phenylalanine, lysine, and histidine. In contrast, the sweet amino acids had the smallest percentage increase of 4%. The increase in glutamic acid in umami amino acids was the most significant at 26.1% among the fresh amino acids, and threonine had the most significant increase of 53.1% among the sweet amino acids. During the YQ stage, the fresh amino acids decreased significantly, while the sweet amino acids increased slightly, and aspartic acid, glutamic acid, arginine, and citrulline decreased more. During the HG stage, there was a significant decrease in citrulline, glutamic acid, arginine, and theanine, which may contribute more to the aroma of chestnut [32].

## 4. Conclusions

Based on HS-SPME and GC–MS technology, all volatile substances in albino tea processing of Longjing tea were separated and identified. Combined with PCA, composition difference analysis, and sensory evaluation, it was found that there were some differences in aroma substances in Longjing tea at different processing stages, and the difference between XY and HG and TX leaves was found to be larger. During the processing of chestnut Longjing tea, the levels of aromatic and floral substances, such as alcohols, aldehydes, and esters, decreased while the levels of aromatic and baking substances, such as ketones, heteroxes, and pyrazines, increased. As a result, the aroma changed from fragrant and floral to pyrogenic, chestnut, and sweet. After conducting a difference comparison and correlation analysis, 2-methylfuran, tetrahydro-3-methyl-5-oxo-2-furancarboxylic acid, 2-((3,3-dimethyloxiran-2-yl)methyl)-3-methylfuran, furfural, 2,2,4,7-tetramethyl-3,5-octanedione, 2-methyl-1-penten-3-one, 3-hexen-2-one, butylated hydroxytoluene, 2-ethyl-butanoic acid-1,2,3-propanetriyl ester, N-caproic acid vinyl ester, nonan-4-yl acetate, dodecane, hexanoyl hexanoate, 1-(1h-pyrrol-2-yl)-ethanone, 2,5-dimethyl-pyrazine, and methyl-pyrazine may be the main aroma-enhancing constituents of chestnut aroma. Processing has a greater effect on the concentration of flavor components in brewed tea than on the absolute content of flavor components in dried tea. This study provides new research value for the processing of the aroma metabolite profile of Longjing tea.

## Data Availability

The original contributions presented in the study are included in the article, further inquiries can be directed to the corresponding authors.

## Supplementary Materials

The following supporting information can be downloaded at https://www.mdpi.com/article/10.3390/foods13091338/s1, Supplemental Table S1: The differentially expressed metabolites of aroma; Supplemental Table S2: Analysis of differential aroma metabolite contents; Supplemental Table S3: Analysis of amino acid compositions in brewed tea soup; Supplemental Table S4: Analysis of amino acid compositions in tea samples; Supplemental Figure S1: The distribution of dry matter contained in tea during different processing processes; and Supplemental Figure S2: The balloon plot of amino acid content change rate in different processing processes.

## References

[1] Yin P., Kong Y.S., Liu P.P., Wang J.J., Zhu Y., Wang G.M., Sun M.F., Chen Y., Guo G.Y., Liu Z.H. (2022). A critical review of key odorants in green tea: Identification and biochemical formation pathway. Trends Food Sci. Technol..

[2] Wang J.Q., Fu Y.Q., Chen J.X., Wang F., Feng Z.H., Yin J.F., Zeng L., Xu Y.Q. (2022). Effects of baking treatment on the sensory quality and physicochemical properties of green tea with different processing methods. Food Chem..

[3] Wang M.Q., Ma W.-J., Shi J., Zhu Y., Lin Z., Lv H.P. (2020). Characterization of the key aroma compounds in Longjing tea using stir bar sorptive extraction (SBSE) combined with gas chromatography-mass spectrometry (GC–MS), gas chromatography-olfactometry (GC-O), odor activity value (OAV), and aroma recombination. Food Res. Int..

[4] Tu Z., Liu Y., Lin J., Lv H., Zhou W., Zhou X., Qian Y., Zeng X., He W., Ye Y. (2023). Comparison of volatile and nonvolatile metabolites in green tea under hot-air drying and four heat-conduction drying patterns using widely targeted metabolomics. Food Chem. X.

[5] Yang Y., Qian M.C., Deng Y., Yuan H., Jiang Y. (2022). Insight into aroma dynamic changes during the whole manufacturing process of chestnut-like aroma green tea by combining GC-E-Nose, GC-IMS, and GC × GC-TOFMS. Food Chem..

[6] Cui H., Mao Y., Zhao Y., Huang H., Yin J., Yu J., Zhang J. (2023). Comparative metabolomics study of four kinds of Xihu Longjing tea based on machine fixing and manual fixing methods. Foods.

[7] Cui J., Zhai X., Guo D., Du W., Gao T., Zhou J., Schwab W.G., Song C. (2022). Characterization of key odorants in xinyang maojian green tea and their changes during the manufacturing process. J. Agric. Food Chem..

[8] Feng Z., Li Y., Li M., Wang Y., Zhang L., Wan X., Yang X. (2019). Tea aroma formation from six model manufacturing processes. Food Chem..

[9] Wang D., Kurasawa E., Yamaguchi Y., Kubota K., Kobayashi A. (2001). Analysis of glycosidically bound aroma precursors in tea leaves. 2. changes in glycoside contents and glycosidase activities in tea leaves during the black tea manufacturing process. J. Agric. Food Chem..

[10] Guo X.Y., Song C., Ho C.T., Wan X. (2018). Contribution of l-theanine to the formation of 2,5-dimethylpyrazine, a key roasted peanutty flavor in Oolong tea during manufacturing processes. Food Chem..

[11] Han Z.X., Rana M.M., Liu G.F., Gao M.J., Li D.X., Wu F.G., Li X.B., Wan X.C., Wei S. (2016). Green tea flavour determinants and their changes over manufacturing processes. Food Chem..

[12] Yang C., Zhou X., Mo X., Guo Y., Hu Y., Gong X. (2017). Dynamic changes of aromatic substances during the formation process of aroma quality in roasted green tea. Guizhou Agric. Sci..

[13] Ni D., Chen Y., Hu J. (1996). Effect of temperature on flavor during second drying of roasted tea. J. Huazhong Agric. Univ..

[14] Shao C.Y., Zhang Y., Lv H.P., Zhang Z.F., Zeng J.M., Peng Q.H., Zhu Y., Lin Z. (2022). Aromatic profiles and enantiomeric distributions of chiral odorants in baked green teas with different picking tenderness. Food Chem..

[15] Yang Z., Baldermann S., Watanabe N. (2013). Recent studies of the volatile compounds in tea. Food Res. Int..

[16] Yin H., Yang Y., Yao Y., Zhang M., Wang J., Jiang Y., Yuan H. (2019). Discrimination of different characteristics of chestnut-like green tea based on gas chromatography-mass spectrometry and multivariate statistical data analysis. Food Sci..

[17] Feng Z., Li Y., Zhang P., Wang J., Xu Y., Feng Y., Zhai X., Yang X., Wan X., Yin J. (2023). Formation and isomerization of (Z)-methyl epijasmonate, the key contributor of the orchid-like aroma, during tea processing. Food Res. Int..

[18] Mizukami Y., Sawai Y., Yamaguchi Y. (2008). Analysis of key odorants in roasted green tea. Tea Res. J..

[19] Wang B., Shu N., Lu A., Liao X., Yan J., Xie G., Tong H. (2020). Aroma formation of green tea effected by different pan-fire temperature. Food Ferment. Ind..

[20] Zhang M., Yin H., Deng Y., Yao Y., Jiang Y., Hua J., Yuan H., Yang Y. (2020). Analysis of key odorants responsible for different chestnut-like aromas of green teas based on headspace solid-phase microextraction coupled with comprehensive two-dimensional gas chromatography time-of-flight mass spectrometry and odor activity value. Food Sci..

[21] An H., Ou X., Zhang Y., Li S., Xiong Y., Li Q., Huang J., Liu Z. (2022). Study on the key volatile compounds and aroma quality of jasmine tea with different scenting technology. Food Chem..

[22] Cui H., Zhang J., Ao C., Huang H., Zheng X., Zhao Y., Shi D., Yu J. (2021). Difference quality characteristics of Xihu Longjing tea with different processing technology. Sci. Technol. Food Ind..

[23] (2013). Tea-Determination of Free Amino Acids Content.

[24] Ye T., Mengxin W., Jinhe W., Baoyu H. (2015). Correlation of low temperature with soluble sugar and amino acid content in fresh tea leaves. J. Tea Sci..

[25] (2018). Methodology for Sensory Evaluation of Tea.

[26] Botelho B.G., Reis N., Oliveira L.S., Sena M.M. (2015). Development and analytical validation of a screening method for simultaneous detection of five adulterants in raw milk using mid-infrared spectroscopy and PLS-DA. Food Chem..

[27] Wang H., Hua J., Jiang Y., Yang Y., Wang J., Yuan H. (2020). Influence of fixation methods on the chestnut-like aroma of green tea and dynamics of key aroma substances. Food Res. Int..

[28] Zhang M., Jiang Y., Yin H., Yang Y., Deng Y., Dong C., Li J., Hua J., Wang J. (2020). Chestnut-like aroma of green tea: Research progress of formation and technology. Chin. Agric. Sci. Bull..

[29] Cui J., Katsuno T., Totsuka K., Ohnishi T., Takemoto H., Mase N., Toda M., Narumi T., Sato K., Matsuo T. (2016). Characteristic fluctuations in glycosidically bound volatiles during tea processing and identification of their unstable derivatives. J. Agric. Food Chem..

[30] Hattori S., Takagaki H., Fujimori T. (2005). A Comparison of the volatile compounds in several green teas. Food Sci. Technol. Int. Tokyo.

[31] Zheng X.Q., Li Q.S., Xiang L.P., Liang Y.R. (2016). Recent advances in volatiles of teas. Molecules.

[32] Ho C.T., Zheng X., Li S. (2015). Tea aroma formation. Food Sci. Hum. Wellness.

[33] Wang M., Zhu Y., Zhang Y., Shi J., Lin Z., Lü H. (2019). Analysis of volatile composition and key aroma compounds of green teas with fresh scent flavor. Food Sci..

[34] Ye Y. (2018). Effects of Withering on the Main Physical and Chemical Properties of Manufactured Tea Leaves.

[35] Ye Y., Yan J., Cui J., Mao S., Li M., Liao X., Tong H. (2018). Dynamic changes in amino acids, catechins, caffeine and gallic acid in green tea during withering. J. Food Compos. Anal..

[36] Huang J., Ding Y., Xu Y., Lei P., Wu Q. (2013). Effects of fresh tea leaves spreading on the quality of green tea. Chin. Tea Process..

[37] Yu X. (2020). Effects of Different Withering Methods on Components Metabolism Related to Color, Aroma and Taste Quality in Green Tea.

