# Supplementary material for: Research of Processing Technology of Longjing Tea with ‘Baiye 1’ Based on Non-Targeted Aroma Metabolomics
Source: Foods. 2024 Apr 26;13(9):1338. doi: 10.3390/foods13091338 (PMC11083364; doi:10.3390/foods13091338)
Supplement: Supplementary file 1 [file foods-13-01338-s001.zip › foods-2923362-supplementary/Supplementary Files/Supplemental Figure S1.pdf]

Supplemental Figure S1: The distribution of dry matter contained in tea during different processing processes.

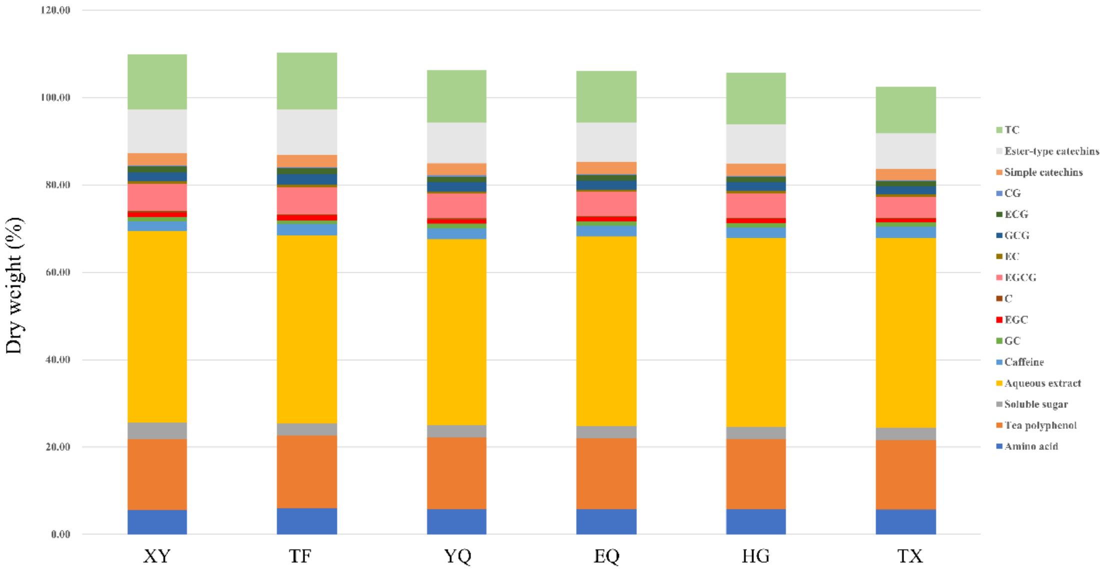

TC, total catechins; GC, gallocatechin; C, catechin; EGC, epigallocatechin; EC, epicatechin; EGCG, epigallocatechin gallate; GCG, gallocatechin gallate; CG, catechingallate; ECG, epicatechin gallate; simple catechins (GC+C+EGC+EC); ester type catechins (EGCG+GCG+CG+ECG); XY indicates fresh leaves, TF indicates spreading fresh leaves, YQ indicates first panning leaves, EQ indicates second panning leaves, HG indicates final panning leaves, TX indicates fragrance enhancing leaves.
