# Supplementary material for: Research of Processing Technology of Longjing Tea with ‘Baiye 1’ Based on Non-Targeted Aroma Metabolomics
Source: Foods. 2024 Apr 26;13(9):1338. doi: 10.3390/foods13091338 (PMC11083364; doi:10.3390/foods13091338)
Supplement: Supplementary file 1 [file foods-13-01338-s001.zip › foods-2923362-supplementary/Supplementary Files/Supplemental Figure S2.pdf]

Supplemental Figure S2: The balloon plot of amino acid content change rate in different processing processes.

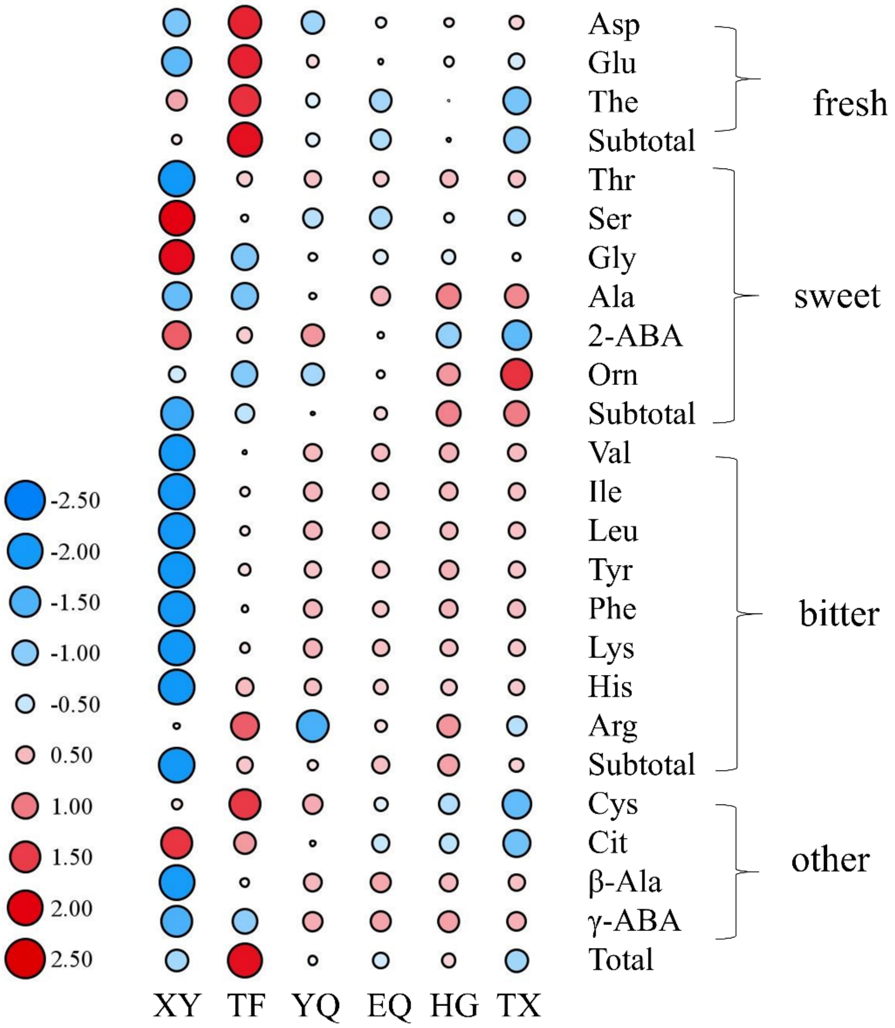

Asp, Aspartic acid; Glu, Glutamic acid; The, theanine; Thr, Threonine; Ser, serine; Gly, glycine; Ala, Alanine; 2-Aba, 2-Aminobutyric acid; Orn, ornithine; Val, valine; Ile, isoleucine; Leu, leucine; Tyr, tyrosine; Phe, phenylalanine; Lys, lysine; His, histidine; Arg, Argnine; Cys, Cysteine; Cit, citrulline; β-Ala, β-alanine, γ-ABA, γ-aminobutyric acid; XY indicates fresh leaves, TF indicates spreading fresh leaves, YQ indicates first panning leaves, EQ indicates second panning leaves, HG indicates final panning leaves, TX indicates fragrance enhancing leaves.
