# Supplementary material for: Research of Processing Technology of Longjing Tea with ‘Baiye 1’ Based on Non-Targeted Aroma Metabolomics
Source: Foods. 2024 Apr 26;13(9):1338. doi: 10.3390/foods13091338 (PMC11083364; doi:10.3390/foods13091338)
Supplement: Supplementary file 1 [file foods-13-01338-s001.zip › foods-2923362-supplementary/Supplementary Files/Supplemental Table S2.pdf]

Supplemental Table S2: Analysis of differential aroma metabolites

Aroma substance	XY	TF	YQ	EQ	HG	TX
Geraniol	10603±3458 Aa	12404±1103 Aa	277±39 Bb	307±36 Bb	269±43 Bb	215±27 Bb
2-Methylfuran	7631±2018 Bb	7482±1063 Bb	11241±461 ABa	12101±1690 ABa	13776±151 Aa	16070±1094 Aa
Linalool	6520±1694 Aa	7478±509 Aa	225±8 Bb	251±30 Bb	210±38 Bb	186±32 Bb
Methyl salicylate	4214±925 Aa	2966±228 Ab	169±45 Bc	135±11 Bc	122±17 Bc	92±8 Bc
Linalool Oxide I	2838±715 Aa	3440±290 Aa	107±7 Bb	144±20 Bb	114±19 Bb	99±17 Bb
Phenylethyl Alcohol	2460±710 Bb	3714±318 Aa	143±38 Cc	138±10 Cc	136±15 Cc	100±6 Cc
β-Myrcene	1368±437 Aa	1483±98 Aa	73.5±8 Bb	87.3±3.8 Bb	74.3±10.7 Bb	71.5±9.4 Bb
Acetaldehyde	862±206 Bb	1721±110 Aa	27.0±2 Cc	27.3±2.6 Cc	25.7±2.2 Cc	24.4±3.6 Cc
1-Hexanol	824±220 Aa	367±24 Bb	15.4±1 Cc	16.8±2.5 Cc	17.5±3.3 Cc	12.7±1.9 Cc
(Z)-3-Hexen-1-ol,	626±168 Aa	486±39 Aa	7.9±0 Bb	7.3±0.8 Bb	7.3±0.8 Bb	5.9±1.0 Bb
(E)-5,9-Undecadien-2-One, 6,10-Dimethyl-,	579±193 Aa	617±53 Aa	8.8±0 Bb	11.6±0.4 Bb	12.9±2.0 Bb	11.2±1.5 Bb
2-Butenal	485±99 Bb	1457±149 Aa	3.1±1 Cc	3.7±0.8 Cc	4.4±1.0 Cc	3.4±1.0 Cc
Benzyl alcohol	480±130 Aa	529±63 Aa	35.6±6 Bb	37.3±3.3 Bb	39.9±4.6 Bb	32.3±3.5 Bb
Trans-β-Ocimene	460±147 Aa	500±37 Aa	23.6±2 Bb	26.5±1.6 Bb	23.0±3.9 Bb	21.9±3.0 Bb
(E)-2-Hexenal	405±111 Aa	399±72 Aa	7.5±1 Bb	8.0±0.9 Bb	11.1±1.8 Bb	9.0±1.5 Bb
Hexanal	395±114 Aa	232±53 Ab	23.4±2 Bc	19.0±3.6 Bc	16.7±2.3 Bc	16.8±4.4 Bc
Carveol	380±98 Ab	538±53 Aa	53.7±4 Bc	88.1±9.8 Bc	83.7±13.8 Bc	81.8±13.2 Bc
(Z)-2,6-Octadien-1-Ol, 3,7-Dimethyl-,	133±39 Bb	237±18 Aa	4.9±1 Cc	5.7±0.4 Cc	4.9±0.9 Cc	4.0±0.2 Cc
Dodecane	44.5±13.1 Bb	46.5±12.3 Bb	46.7±2.1 Bb	49.9±5.6 Bb	148.2±27.5 Aa	172.6±34.2 Aa
2-Ethyl-Butanoic Acid, 1,2,3-Propanetriyl Ester	33.8±9.4 Cd	47.6±5.3 Cd	257.8±8.6 Bc	346.2±41.5 ABab	391.1±49.9 Aa	279.9±40.9 ABbc
N-Caproic Acid Vinyl Ester	19.8±6.8 Bb	18.9±1.4 Bb	90.5±5.1 Aa	107.5±15.1 Aa	117.6±26.1 Aa	91.6±22.6 Aa
2-Furancarboxylic Acid, Tetrahydro-3-Methyl-5-Oxo-	9.44±1.98 Cc	13.6±1.7 Cc	85.4±3.7 Bb	114.4±13.6 ABab	130.7±18.4 Aa	92.6±13.2 ABb
3,5-Octanedione, 2,2,4,7-Tetramethyl-	3.3±0.58 Cc	4.00±0.53 Cc	75.9±6.2 Bb	105.3±13.9 ABab	126.1±17.4 Aa	105.8±21.3 ABab
Hexanoic Acid, Anhydride	3.04±0.92 Cc	3.86±0.49 Cc	31.12±1.33 Bb	38.68±4.16 ABab	44.56±5.82 ABa	33.88±4.27 Ab
Butylated Hydroxytoluene	2.61±0.56 Cc	3.20±0.29 Cc	7.01±0.98 Cc	13.32±1.04 Bb	17.85±2.27 Bb	27.48±3.18 Aa
Nonan-4-Yl Acetate	2.54±0.90 Cc	3.46±0.36 Cc	22.17±0.76 Bb	28.34±2.81 ABab	32.81±3.73 Aa	24.73±3.33 ABb
1-(1h-Pyrrol-2-Yl)- Ethanone	2.35±1.42 Bb	1.58±0.23 Bb	6.98±0.80 Bb	21.22±2.91 Aa	24.05±4.20 Aa	25.06±2.44 Aa
2-((3,3-Dimethyloxiran-2-Yl) Methyl)-3-Methylfuran	1.20±0.42 Bc	2.16±0.80 Bbc	4.12±0.09 Bb	10.62±0.82 Aa	10.68±1.09 Aa	11.99±1.67 Aa
Furfural	0.96±0.50 Bc	2.61±2.40 Bc	4.90±0.24 ABbc	8.99±0.47 Aab	8.48±0.61 Aab	10.16±2.62 Aa
Pyrazine, 2,5-Dimethyl-	0.85±0.44 Cc	0.93±0.25 Cc	17.71±0.56 Bb	35.99±2.08 Aa	38.01±6.67 Aa	31.91±3.77 Aa
1-Penten-3-One, 2-Methyl-	0.65±0.51 Bc	0.50±0.15 Bc	3.09±0.28 Bb	5.91±0.43 Aa	7.96±1.26 Aa	7.36±1.31 Aa
3-Hexen-2-One	0.34±0.04 Bb	2.39±1.85 Bb	10.48±0.47 Bb	38.55±3.52 Aa	42.74±8.13 Aa	45.89±6.07 Aa
Pyrazine, Methyl-	0.11±0.12 Cc	0.31±0.19 Cc	7.82±0.60 Bb	13.46±0.60 Aa	14.88±2.24 Aa	14.68±2.49 Aa

μg/kg tea leaves. Different uppercase letters indicate $p<0.01$ level and different lowercase letters indicate $p<0.05$.
