# Supplementary material for: Research of Processing Technology of Longjing Tea with ‘Baiye 1’ Based on Non-Targeted Aroma Metabolomics
Source: Foods. 2024 Apr 26;13(9):1338. doi: 10.3390/foods13091338 (PMC11083364; doi:10.3390/foods13091338)
Supplement: Supplementary file 1 [file foods-13-01338-s001.zip › foods-2923362-supplementary/Supplementary Files/Supplemental Table S3.pdf]

Supplemental Table S3: Analysis of amino acid compositions in brewed tea soup

Types	Components	XY	TF	YQ	EQ	HG	TX
Fresh	L-aspartic acid	19.2±0.3 CDd	17.2±0.2 Dd	23.5±1.0 Cc	40.4±0.6 Bb	59.7±1.0 Aa	62.9±2.7 Aa
	L-glutamic acid	26.5±0.5 Dd	25.0±0.3 Dd	36.3±1.8 Cc	60.7±1.2 Bb	84.4±1.6 Aa	86.7±0.7 Aa
	L-theanine	174.7±3.8 CDc	132.5±0.1 Dd	183.1±6.9 Cc	343.8±14.0 Bb	614.6±27.8 Aa	642.0±4.2 Aa
	subtotal	220.4±4.6 CDc	174.8±0.6 Dd	242.8±9.6 Cc	444.9±15.7 Bb	758.7±30.4 Aa	791.6±1.0 Aa
Sweet	L-threonine	1.75±0.02 De	2.12±0.03 Dd	3.56±0.17 Cc	5.84±0.07 Bb	8.63±0.12 Aa	8.96±0.18 Aa
	L-serine	6.9±0.1 De	5.1±0.0 Ee	8.3±0.4 Cc	13.3±0.1 Bb	19.0±0.6 Aa	19.9±0.1 Aa
	L-glycine	0.40±0.01 De	0.27±0.01 Ef	0.45±0.01 Cd	0.72±0.01 Bc	1.00±0.01 Ab	1.05±0.03 Aa
	L-alanine	2.9±0.1 Dd	2.2±0.0 Ee	3.9±0.2 Cc	6.0±0.1 Bb	8.5±0.3 Aa	8.9±0.2 Aa
	2-Aminobutyric acid	0.13±0.00 CDd	0.11±0.01 Dd	0.18±0.02 Cc	0.33±0.02 Bb	0.45±0.02 Aa	0.45±0.04 Aa
	L-methionine	0.04±0.00 De	0.04±0.00 De	0.10±0.00 Cd	0.18±0.00 Bc	0.29±0.01 Ab	0.31±0.01 Aa
	L-ornithine	0.76±0.02 Dd	0.64±0.01 Dd	0.80±0.05 Dd	1.54±0.08 Cc	2.59±0.03 Bb	2.97±0.15 Aa
	subtotal	12.9±0.2 De	10.5±0.1 Ef	17.3±0.8 Cd	27.8±0.2 Bc	40.5±1.0 Ab	42.5±0.2 Aa
Bitter	L-valine	0.49±0.02 Ee	1.27±0.02 Dd	2.20±0.03 Cc	3.10±0.13 Bb	4.01±0.06 Aa	4.05±0.03 Aa
	L-isoleucine	0.12±0.01 Ef	0.62±0.00 De	1.17±0.03 Cd	1.78±0.06 Bc	2.51±0.01 Ab	2.62±0.00 Aa
	L-leucine	0.35±0.03 Ff	1.39±0.01 Ee	2.50±0.07 Dd	3.80±0.08 Cc	5.33±0.05 Bb	5.57±0.05 Aa
	L-tyrosine	0.15±0.01 Ef	0.72±0.01 De	1.28±0.03 Cd	2.27±0.04 Bc	4.32±0.06 Ab	4.45±0.04 Aa
	L-phenylalanine	0.32±0.02 Ee	1.88±0.03 Dd	3.47±0.16 Cc	6.24±0.27 Bb	10.96±0.12 Aa	11.42±0.30 Aa
	L-lysine	0.18±0.02 Ff	0.47±0.00 Ee	1.24±0.08 Dd	2.41±0.08 Cc	4.23±0.00 Bb	4.45±0.04 Aa
	L-histidine	0.32±0.02 Dd	0.36±0.01 Dd	0.83±0.04 Cc	1.66±0.08 Bb	2.85±0.14 Aa	3.00±0.26 Aa
	L-arginine	10.6±0.1 CDcd	6.4±0.0 Dd	14.6±0.6 Cc	30.9±2.2 Bb	59.0±3.3 Aa	63.3±0.8 Aa
	subtotal	12.5±0.2 Dd	13.1±0.1 Dd	27.3±1.0 Cc	52.2±2.9 Bb	93.2±3.5 Aa	98.9±0.7 Aa
Other	L-cystine	3.9±0.1 Dd	3.3±0.0 Dd	5.2±0.1 Cc	9.1±0.1 Bb	15.1±0.2 Aa	15.7±0.9 Aa
	L-citrulline	0.40±0.02 Cc	0.26±0.01 Dd	0.43±0.01 Cc	0.72±0.06 Bb	1.14±0.04 Aa	1.13±0.03 Aa
	β-Alanine	0.09±0.00 Ee	0.19±0.01 Dd	0.37±0.02 Cc	0.58±0.015 Bb	0.89±0.01 Aa	0.93±0.01 Aa
	3-aminoisobutyric acid	0.061±0.012 Ccd	0.06±0.00 Cd	0.09±0.01 Cc	0.14±0.01 Bb	0.29±0.01 Aa	0.30±0.00 Aa
	γ-aminobutyric acid	0.59±0.01 Dd	0.59±0.02 Dd	1.79±0.11 Cc	2.60±0.09 Bb	4.02±0.26 Aa	4.10±0.15 Aa
	total	250.9±5.1 CDd	202.8±0.7 Dd	295.4±11.6 Cc	538.0±19.1 Bb	913.9±35.5 Aa	955.2±0.8 Aa

μg/ml in brewed tea soup. Different uppercase letters indicate $p<0.01$ level and different lowercase letters indicate $p<0.05$.
