# Supplementary material for: Research of Processing Technology of Longjing Tea with ‘Baiye 1’ Based on Non-Targeted Aroma Metabolomics
Source: Foods. 2024 Apr 26;13(9):1338. doi: 10.3390/foods13091338 (PMC11083364; doi:10.3390/foods13091338)
Supplement: Supplementary file 1 [file foods-13-01338-s001.zip › foods-2923362-supplementary/Supplementary Files/Supplemental Table S4.pdf]

Supplemental Table S4: Analysis of amino acid compositions in tea samples

Types	Components	XY	TF	YQ	EQ	HG	TX
Fresh	L-aspartic acid	292.3±1.6 Dd	333.0±1.8 Aa	296.6±3.3 Dd	306.2±1.7 Cc	310.5±0.4 BCbc	313.0±0.7 Bb
	L-glutamic acid	455.1±2.0 Ee	573.7±1.6 Aa	517.6±2.0 Bb	506.5±2.1 Cc	502.4±1.0 Cc	493.1±2.4 Dd
	L-theanine	4202.1±78.2 ABb	4330.4±13.6 Aa	4065.9±74.6 BCcd	3997.9±24.4 Ccd	4108.4±14.8 BCbc	3945.4±16.3 Cd
	subtotal	4949.5±78.4 Bb	5237.0±13.7 Aa	4880.1±79.9 BCbc	4810.6±28.1 BCbc	4921.3±14.4 BCb	4751.4±17.7 Cc
Sweet	L-threonine	31.8±0.1 Cd	48.7±0.3 Bc	49.4±0.3 ABab	48.8±0.4 ABbc	49.7±0.1 Aa	49.3±0.2 ABabc
	L-serine	117.1±0.2 Aa	111.1±0.3 Bb	109.5±1.1 Bbc	109.2±0.5 Bc	110.9±0.3 Bbc	110.1±0.9 Bbc
	L-glycine	6.7±0.3 Aa	5.2±0.1 Bb	5.7±0.1 Bb	5.6±0.2 Bb	5.6±0.3 Bb	5.7±0.1 Bb
	L-alanine	42.4±0.2 Dd	43.0±0.2 Dd	47.4±0.5 Cc	49.2±0.2 Bb	50.6±0.1 Aa	50.4±0.3 Aa
	2-Aminobutyric acid	2.7±0.1 Aa	2.5±0.1 ABab	2.6±0.1 Aab	2.4±0.1 ABCbc	2.2±0.1 BCcd	2.1±0.0 Cd
	L-ornithine	17.2±0.1 CDcd	15.8±0.4 Ee	16.4±0.2 DEde	17.9±0.5 Cc	19.8±0.2 Bb	21.5±0.1 Aa
	subtotal	216.9±1.6 Dd	226.2±0.3 Cc	231.0±1.9 Bb	233.1±0.4 Bb	238.8±0.3 Aa	239.1±1.5 Aa
Bitter	L-valine	8.6±0.0 Cc	25.6±0.2 Bb	30.1±0.1 Aa	29.9±0.9 Aa	30.7±0.2 Aa	30.0±0.4 Aa
	L-isoleucine	2.2±0.1 Dd	14.5±0.3 Cc	16.8±0.3 Aa	16.1±0.3 Bb	16.6±0.0 ABab	16.3±0.0 ABb
	L-leucine	5.6±0.3 Dd	31.7±0.1 Cc	36.5±0.1 Aa	35.3±0.3 Bb	35.9±0.2 ABab	35.5±0.5 ABb
	L-tyrosine	4.2±0.2 Cd	25.8±0.2 Bc	27.7±0.7 Ab	27.7±0.6 Aab	28.9±0.3 Aa	27.6±0.3 Ab
	L-phenylalanine	7.6±0.1 Dd	56.1±0.4 Cc	66.8±1.2 Aa	63.5±1.4 Bb	67.0±0.3 Aa	65.7±0.3 ABa
	L-lysine	8.2±0.3 Cd	31.5±0.6 Bc	35.8±0.1 Aa	34.5±0.5 Ab	34.8±0.4 Aab	34.3±0.5 Ab
	L-histidine	9.9±0.4 Bb	17.7±0.4 Aa	17.6±0.8 Aa	17.2±0.4 Aa	17.4±0.4 Aa	17.4±0.1 Aa
	L-arginine	508.8±3.4 Bc	530.6±1.7 Aa	483.3±1.0 De	513.4±1.5 Cc	523.2±2.4 Ab	499.4±2.8 Cd
	subtotal	555.1±3.1 De	733.5±2.0 Bbc	714.5±0.6 Cd	737.7±5.3 Bb	754.4±3.5 Aa	726.2±4.4 BCc
Other	L-cystine	107.4±1.2 Cc	117.7±0.3 Aa	110.9±1.0 Bb	104.1±0.1 Dd	101.2±0.7 Ee	96.1±0.7 Ff
	L-citrulline	9.9±0.2 Aa	9.0±0.1 Bb	8.1±0.1 Cc	7.6±0.1 Dd	7.5±0.0 Dd	6.8±0.1 Ee
	β-Alanine	1.79±0.05 Cc	3.51±0.38 Bb	4.09±0.11 ABa	4.20±0.21 Aa	4.08±0.10 ABa	4.03±0.07 ABab
	γ—aminobutyric acid	8.8±0.1 Dd	12.8±0.2 Cc	24.1±0.3 ABab	24.5±0.3 ABab	24.7±0.2 Aa	23.8±0.3 Bb
	total	5850.3±74.9 Dd	6339.7±15.2 Aa	5972.8±82.8 BCbc	5921.7±33.6 BCDbc	6052.1±16.5 Bb	5847.4±24.2 CDcd

mg/100g (dry matter rate). Different uppercase letters indicate $p<0.01$ level and different lowercase letters indicate $p<0.05$.
